# Evaluation of the role of metabolizing enzymes and transporter variants in ezetimibe pharmacokinetics

**DOI:** 10.3389/fphar.2024.1414059

**Published:** 2024-10-17

**Authors:** Eva González-Iglesias, Dolores Ochoa, Marcos Navares-Gómez, Pablo Zubiaur, Marina Aldama, Tamara de la Torre, Marta de los Ríos-Rodríguez, Paula Soria-Chacartegui, Andrea Rodríguez-Lopez, Francisco Abad-Santos, Jesús Novalbos

**Affiliations:** ^1^ Clinical Pharmacology Department, Hospital Universitario de La Princesa, Instituto de Investigación Sanitaria La Princesa (IIS-Princesa), Madrid, Spain; ^2^ Pharmacology Department, Faculty of Medicine, Universidad Autónoma de Madrid (UAM), Madrid, Spain; ^3^ Centro de Investigación Biomédica en Red de Enfermedades Hepáticas y Digestivas (CIBERehd), Instituto de Salud Carlos III, Madrid, Spain

**Keywords:** ezetimibe, simvastatin, pharmacogenetics, pharmacokinetics, bioequivalence trials

## Abstract

**Introduction:**

Ezetimibe inhibits cholesterol uptake by modulation of intestinal sterol absorption. Currently, although some studies have shown alterations in ezetimibe levels caused by alterations in the *ABCG5*, *ABCG8*, *NPC1L1* or *UGT1A1* genes, there are no pharmacogenetic guidelines to confirm these biomarkers. The aim of this work was to evaluate the effect of 49 variants in 22 pharmacogenes related to metabolism and transport.

**Methods:**

A total of 96 healthy volunteers from four bioequivalence clinical trials of ezetimibe as monotherapy or in combination with simvastatin were studied. Blood samples were extracted for unconjugated ezetimibe plasma quantification and genotyping.

**Results and Discussion:**

No association of metabolizing enzyme variants with ezetimibe pharmacokinetic parameters was found. The results show some trends in the univariate analysis for *ABCB1* rs2032582 or *ABCC2* rs2273697 and C_max_ (*p univariate* (*p*_*uv*_) = 0.056 and 0.087, respectively), which finally reach significance in the multivariate analysis (*p multivariate* (*p*_*mv*_) = 0.049 and 0.048, respectively). Nevertheless, these results need to be validated in future studies.

## 1 Introduction

The leading cause of mortality worldwide is cardiovascular disease, with high levels of blood cholesterol being one of the main contributors (Zhan et al., 2018). To improve this situation, a group of drugs called statins were developed, which became the first-line lipid-lowering drugs and the most widely prescribed (Boutari et al., 2021). Statins inhibit the enzyme responsible for the rate-limiting step in cholesterol biosynthesis, HMG-CoA reductase. A large number of patients do not achieve target levels of low-density lipoprotein cholesterol (LDL-C), even at the maximum tolerated dose of statins. For this reason, combination therapy with drugs such as ezetimibe is often prescribed (Hirota et al., 2020).

Ezetimibe was developed as a potential inhibitor of intracellular acyl-coenzyme A cholesterol acyltransferase (ACAT), but was ultimately demonstrated to inhibit cholesterol uptake (Gryn and Hegele, 2015). This occurs through modulation of intestinal sterol absorption by interaction with aminopeptidase N and inhibition of the sterol transporter Niemann-Pick C1-like protein (NPC1L1) in the small intestine and liver (Schmitz et al., 2007). Statin treatment combined with 10 mg of ezetimibe results in a 15%–25% reduction in incremental LDL-C compared to monotherapy (Schmitz et al., 2007; Ferreira and Marques da Silva, 2017; Tada et al., 2020; Kei et al., 2016).

Ezetimibe undergoes extensive first-pass metabolism in intestinal wall cells mediated by UDP-glucuronosyltransferases (UGT) 1A1 (UGT1A1) and 1A3 (UGT1A3) and, to a lesser extent, 2B15 (UGT2B15). This results in the pharmacologically active metabolite, ezetimibe glucuronide, which accounts for approximately 80%–90% of the total drug in plasma (Schmitz et al., 2007; Soulele and Karalis, 2019; Oswald et al., 2006). Ezetimibe glucuronide reaches its maximum plasma concentration (C_max_) between 1 and 2 h, and ezetimibe between 4 and 12 h after administration. After conjugation, the active metabolite binds with high affinity to NPC1L1 to prevent cholesterol absorption (Oswald et al., 2006).

Both single and ezetimibe glucuronide have multiple peaks in their plasma concentration-time profiles due to enterohepatic recirculation. This means that their elimination from the blood does not follow the usual process. This recirculation involves both transport from the intestine via the P-glycoprotein (coded by *ABCB1* gene) and into the liver via the portal vein. Hepatic uptake is mediated by OATP1B1 (encoded by the *SLCO1B1* gene) and subsequently ezetimibe is glucuronidated and secreted through the gallbladder back into the intestine via the ABCC2 transporter (Oswald et al., 2011). Ezetimibe glucuronide undergoes enzymatic hydrolysis in the intestinal lumen and is converted back to the original drug, which is reabsorbed into the systemic circulation. This process continues until the drug is completely eliminated from the body. Seventy-eight percent of ezetimibe is excreted in the feces and 11% in the urine, with a half-life of 22 h for both ezetimibe and ezetimibe glucuronide (Soulele and Karalis, 2019; Jeu and Cheng, 2003; Ministerio de Sanidad and Política Social e Igualdad, 2013). Enterohepatic recirculation gives ezetimibe great potential to increase its cholesterol-lowering activity by prolonging the time the drug spends in the intestinal lumen (Soulele and Karalis, 2019; Jeu and Cheng, 2003).

Genetic variation in genes involved in the metabolism or transport of drugs (i.e., pharmacogenes) may modify their safety and/or efficacy. Currently, although some studies have shown alterations in ezetimibe levels caused by alterations in the *ABCG5*, *ABCG8*, *NPC1L1* or *UGT1A1* genes, there are no pharmacogenetic guidelines to confirm these biomarkers (Tada et al., 2020; Bae et al., 2011; Calderon-Ospina et al., 2020; Hegele et al., 2005; Zambrano et al., 2015). The aim of this study was to evaluate the effect of 49 genetic variants in 22 pharmacogenes related to drug metabolism and transport on the pharmacokinetics of ezetimibe in healthy volunteers. The present work is part of the La Princesa Multidisciplinary Initiative for the Implementation of Pharmacogenetics (PriME-PGx) (Zubiaur et al., 2021a).

## 2 Material and methods

### 2.1 Study population

A total of 96 healthy volunteers who participated in four bioequivalence trials of ezetimibe in monotherapy or in combination with simvastatin were included in this research. The studies were conducted at the Clinical Trials Unit of Hospital Universitario La Princesa (UECHUP), Madrid (Spain) (https://www.iis-princesa.org/infraestructuras/ensayos-clinicos/informacion-para-promotores/) in 2015 and 2016 (EUDRA-CT numbers: 2014–005512–42 (A), 2015–002393–20 (B), 2015–005778–39 (C), 2016–004211–12 (D).

The inclusion criteria were: males or females aged from 18 to 55, free from organic or psychic conditions, with normal medical records, vital signs, electrocardiogram and physical examination and without significant abnormalities in hematology, coagulation, biochemistry, serology and urine analysis. The exclusion criteria were: having received medication within 2 days prior to the start of the study, having a body mass index (BMI) outside the 18.5–30.0 range, being pregnant or breastfeeding women, having history of sensitivity to any drug, having a positive drug screening, smoking or alcoholism, blood donation in the last month and participation in another study with investigational drugs in the three previous months.

All the clinical trials were approved by the Spanish Drugs Agency (AEMPS) and the Research Ethics Committee Board (CEIm) of the Hospital Universitario de La Princesa. The development of the trials and the handling of data were conducted in compliance with Spanish Legislation, the International Council on Harmonization (ICH) guidelines on Good Clinical Practice (European Medicines Agency, 2024), the ezetimibe product-specific bioequivalence guidance (Committee for Medicinal Products for Human Use CHMP, 2019) and the Revised Declaration of Helsinki (World Medical Association Declaration of Helsinki, 2013). Healthy volunteers signed an informed consent to participate in the pharmacogenetic study (code SFC-FG-2020–1, IRB/Code: 4,176) that was evaluated by the IRB/EC board of Hospital Universitario La Princesa and approved on 9 July 2020. Finally, 96 gave written consent to participate.

### 2.2 Study design and procedures

Data were collected from four bioequivalent trials whose objective was the comparison of a test and a reference formulation of ezetimibe in monotherapy or in combination with simvastatin. All the clinical trials were open, randomized, crossover and with two periods and two sequences. Clinical trials A and B evaluated a single oral dose of ezetimibe 10 mg and clinical trials C and D evaluated a single oral dose of ezetimibe/simvastatin 10/40 mg. Only data from the reference formulation was analyzed in this study. Subject were hospitalized from 10 h previous to 24 h after drug intake. At least 16 blood samples were extracted from pre-dose to 72 h after drug intake in each period for ezetimibe quantification.

### 2.3 Pharmacokinetic analysis

Unconjugated ezetimibe plasma concentration was measured by an external laboratory by high performance liquid chromatography with mass spectrometry (HPLC-MS/MS). Analytical determination of ezetimibe and ezetimibe/simvastatin was blinded. The lower limit of quantification was established at 40 pg/mL. The concentration values obtained were used to calculate pharmacokinetic parameters with WinNonLin Professional Software version 6.3 (Scientific Consulting, Inc., Cary, NC, United States) for clinical trials A and C, version 6.4 (Scientific Consulting, Inc., Cary, NC, United States) for clinical trial B and version 7 (Scientific Consulting, Inc., Cary, NC, United States) for clinical trial D. The area under the curve (AUC) from pre-dose to 72 h (AUC_72h_) was calculated using the plasmatic concentrations according to the linear trapezoidal rule. The C_max_ and time to C_max_ (t_max_) parameters were obtained directly from the concentration-time curve. Half-life determination is hampered due to the enterohepatic recirculation, thus, it was excluded from the analysis.

### 2.4 Genotyping

DNA from blood samples was extracted using a MagNA Pure instrument (Roche Applied Science, United States) or a Maxwell^®^ RSC Automated DNA extractor (Promega Biotech Iberica S.L). Genotyping was performed by a QuantStudio 12 K Flex qPCR instrument with an OpenArray thermal block (Applied Biosystems, Thermofisher, United States) using a custom array described in Zubiaur et al., that includes variants for metabolizing enzymes and transporters (Zubiaur et al., 2021a). Table 1 shows the variants analyzed in this study and the gene to which they correspond. Techniques were carried out in the pharmacogenetics unit of the Clinical Pharmacology Department of the Hospital Universitario de La Princesa.

**TABLE 1:** Genotyped variants and alleles in which those variants are present.

Gene	Allele	Variants present in the allele	European allele frequency	Latin-american allele frequency
*ABCB1*	N/A	rs1128503	0.57	0.52
	N/A	rs1045642	0.48	0.55
	N/A	rs2032582^a^	0.55 (C), <0.01(T)	0.54 (C), <0.01(T)
*ABCC2*	N/A	rs2273697	0.20	0.16
*SLC22A1*	N/A	rs72552763	0.15	0.27
	N/A	rs12208357	0.07	0.02
	N/A	rs34059508	0.02	0.01
*SLCO1B1*	*5	rs4149056^b^	0.02	0.00
	*15	rs4149056, rs2306283^b^	0.15	0.24
	*37	rs2306283^b^	0.25	0.39
*CYP1A2*	N/A	rs2470890	0.35	0.66
	N/A	rs2069514	0.01	0.34
	N/A	rs762551	0.70	0.69
*CYP2A6*	*9	rs28399433	0.06	0.04
*CYP2B6*	*4	rs2279343	0.04	0.11
	*5	rs3211371	0.12	0.04
	*6	rs2279343, rs3745274	0.23	0.21
	*7	rs2279343, rs3211371, rs3745274	0.02	0.01
	*9	rs3745274	0.01	0.07
*CYP2C19*	*2	rs4244285	0.15	0.10
	*3	rs4986893	<0.01	<0.01
	*4	rs28399504	<0.01	<0.01
	*17	rs12248560	0.22	0.17
*CYP2C8*	*2	rs11572103	<0.01	<0.01
	*3	rs10509681	0.12	0.08
	*4	rs1058930	0.04	0.01
*CYP2C9*	*2	rs1799853	0.13	0.08
	*3	rs1057910	0.08	0.04
*CYP2D6*	*3	rs35742686	0.02	0.01
	*4	rs3892097, rs1065852	0.19	0.12
	*6	rs5030655	0.01	0.01
	*7	rs5030867	<0.01	0.00
	*8	rs5030865	<0.01	0.00
	*9	rs5030656	0.03	0.02
	*10	rs1065852	0.02	0.03
	*14	rs5030865	0.00	0.00
	*17	rs28371706	<0.01	0.02
	*41	rs28371725	0.09	0.05
*CYP3A4*	*2	rs55785340	<0.01	0.00
	*6	rs4646438	<0.01	0.00
	*22	rs35599367	0.05	0.03
*CYP3A5*	*3	rs776746	0.92	0.77
	*6	rs10264272	<0.01	0.04
*UGT1A1*	*80	rs887829	0.31	0.38
*UGT1A3*	N/A	rs2008584	0.44	0.47
*UGT1A4*	N/A	rs2011425	0.08	0.01
*UGT1A6*	N/A	rs10445704	0.40	0.27
*UGT1A8*	N/A	rs1042597	0.22	0.02
*UGT1A9*	N/A	rs10929302	0.29	0.30
*UGT2B7*	N/A	rs7668258	0.48	0.71
*UGT2B10*	N/A	rs61750900	0.07	0.00
*UGT2B15*	N/A	rs1902023	0.48	0.61

N/A: not applicable. Allelic frequency data was collected from the Clinical Pharmacogenetics Implementation Consortium (CPIC) and the National Institutes of Health (NIH) SNP, databases.

^a^ As this is a triallelic variant, the allele frequencies for the two alternatives are shown.

^b^ As allelic references for Latin Americans are not available in CPIC, those for Americans are reflected globally.

### 2.5 Phenotyping and haplotyping

Genotype-informed phenotypes for metabolizing enzymes or transporters were inferred according to the Clinical Pharmacogenetics Implementation Consortium (CPIC) or the Dutch Pharmacogenetic Working Group (DPWG) guidelines for the following genes: *CYP2B6* (Bousman et al., 2023), *CYP2C19* (Bousman et al., 2023), *CYP2C9* (Cooper-DeHoff et al., 2022), *CYP2D6* (Bousman et al., 2023), *CYP3A5* (Birdwell et al., 2015), *SLCO1B1* (Cooper-DeHoff et al., 2022) and *UGT1A1* (Gammal et al., 2016). Since *CYP2D6* is not one of the main candidate genes, copy number variants (CNVs) were not performed because they would require more costs than expected benefits. Phenotypes were classified in ultrarapid, rapid, normal, intermediate and poor metabolizers (UM, RM, NM, IM, PM, respectively) for metabolizing enzymes (Bousman et al., 2023; Cooper-DeHoff et al., 2022; Birdwell et al., 2015; Gammal et al., 2016). In the case of transporters, the function was classified as increased, normal, intermediate or poor function (IF, NM, DF, PF, respectively) (Cooper-DeHoff et al., 2022). *CYP2C8* phenotype was establish as previously described by Campodonico *et al.* in 2022 (Campodónico et al., 2022). The remaining variants were analyzed individually.

### 2.6 Statistical analysis

SPSS software (version 23, SPSS Inc., Chicago, IL, United States) was used to perform the statistical analysis. AUC_72h_ and C_max_ parameters were divided by the dose/weight ratio (DW) to correct the effect of dose and weight, the latter especially in order to control for the effect of weight differences between men and women on pharmacokinetic variability. Pharmacokinetic parameters were analyzed according to sex, biogeographical origin, clinical trial, co-administration of drugs, genotypes and phenotypes. Variable distributions were checked for normality with a Shapiro–Wilks test. Variables not normally distributed were logarithmically transformed and normality was re-evaluated. For the pharmacokinetic variables following a normal distribution with two categories, a *t*-test was performed, whereas an ANOVA test followed by the Bonferroni *post hoc* was applied for those with three or more categories. For those not following a normal distribution, non-parametric tests were used. A Mann–Whitney test was used for variables with two categories and a Kruskal–Wallis test for those with three or more categories. The *p*-value considered for statistically significant associations was *p* < 0.05. The multivariate analysis was performed by means of linear regression, in which those independent genetic variables with a *p-*value lower than 0.1 in the univariate analysis were included along with sex, biogeographical origin and clinical trial.

## 3 Results

### 3.1 Demographic characteristics

Study population was composed of 51 men (53%) and 45 women (47%) (Table 2). Height, weight and BMI were significantly lower in women than in men (*p* < 0.001). European was the most prevalent biogeographical origin (83%) compared to Latin-American (14%). Since only two volunteers were self-identified as East Asian and one as Near Eastern, they were merged into a single group under the name “Other”. No differences in age, weight, height and BMI were observed according to clinical trial (Table 2).

**TABLE 2:** Demographic characteristics regarding sex, biogeographical origin and clinical trial.

	N	Age (years)	Weight (kg)	Height (m)	BMI (kg/m^2^)
Sex					
Men	51	23.00 (22.00–28.00)	76.18 (8.90)	1.77 (0.06)	24.21 (22.58–26.23)
Women	45	24.00 (22.00–26.00)	58.19 (7.71)^b^	1.64 (0.07)^b^	20.89 (20.43–22.41)^b^
Biogeographical Origin					
European	80	23.50 (22.00–26.00)	68.49 (12.42)	1.71 (0.09)	22.45 (20.73–25.32)
Latin-American	13	23.00 (22.00–41.00)	64.89 (11.55)	1.69 (0.10)	22.59 (20.23–24.06)
Other^a^	3	24.00 (19.00–24.00)^c^	60.17 (10.15)	1.62 (0.05)	23.74 (19.43–25.08)^c^
Clinical Trial					
A	33	23.00 (22.00–25.00)	67.98 (11.62)	1.72 (0.10)	23.04 (20.71–24.22)
B	11	24.00 (22.00–30.00)	65.52 (10.59)	1.70 (0.09)	22.58 (20.68–25.08)
C	30	23.00 (22.00–24.25)	67.70 (12.13)	1.72 (0.09)	22.48 (20.84–24.47)
D	22	26.00 (23.25–31.00)	68.58 (14.69)	1.69 (0.10)	22.45 (20.40–27.35)

Data are shown as median (Q25-Q75) for non-normal distributions and mean (standard deviation) for normal distributions. BMI: body mass index.

^a^ Other: one volunteer self-reported as Near Eastern and two as East Asian.

^b^ *p* < 0.05 compared to men.

^c^ It is not possible to generate quartiles, therefore, the range of the data is shown for this result.

### 3.2 Pharmacokinetics

Ezetimibe mean AUC_72h_ was 110.84 ± 49.05 h*ng/mL (93.25 ± 35.95 h*ng/mL for men and 130.78 ± 54.41 h*ng/mL for women, *p* < 0.001); and mean C_max_ was 5.35 ± 3.01 ng/mL (4.40 ± 1.90 ng/mL for men and 6.42 ± 3.65 ng/mL for women, *p* = 0.001). After DW correction, the differences between men and women disappeared (Table 3). No differences in pharmacokinetic parameters were observed according to biogeographical origin, clinical trial or co-administration of drugs (Table 3).

**TABLE 3:** Ezetimibe pharmacokinetic parameters based on sex, biogeographical origin, clinical trial and co-administration of drugs.

	N	AUC_72h_/DW (h^a^ng^a^kg/mL^a^mg)	C_max_/DW (ng^a^kg/mL^a^mg)	t_max_ (h)
Sex				
Men	51	702.49 (263.96)	33.24 (14.65)	5.50 (1.25–6.50)
Women	45	750.95 (305.26)	37.07 (20.93)	5.50 (1.50–6.00)
Biogeographical Origin				
European	80	737.55 (289.10)	35.84 (18.83)	5.50 (1.50–6.00)
Latin-American	13	639.96 (232.90)	31.03 (12.67)	6.00 (0.50–10.25)
Other^b^	3	765.45 (370.58)	31.01 (7.51)	1.50 (1.50–2.50)^a^
Clinical Trial				
A	33	737.17 (316.01)	32.50 (17.13)	6.00 (1.50–6.50)
B	11	743.27 (320.04)	31.62 (7.98)	6.00 (1.50–6.50)
C	30	666.81 (252.05)	36.24 (17.44)	2.50 (1.00–6.00)
D	22	777.86 (258.77)	38.91 (22.68)	5.50 (1.69–6.13)
Co-administration of drugs				
Ezetimibe	44	738.69 (313.28)	32.28 (15.28)	6.00 (1.50–6.50)
Ezetimibe/Simvastatin	52	713.80 (258.39)	37.37 (19.66)	3.00 (1.25–6.00)

Data are shown as mean (standard deviation) for normal distributions and median (Q25-Q75) for non-normal distributions. AUC: area under the curve. DW: dose/weight ratio. C_max_: maximum concentration. T_max_: time to C_max_.

^a^ It is not possible to generate quartiles, therefore, the range of the data is shown for this result.

^b^ Other: one volunteer self-reported as Near Eastern and two as East Asian.

### 3.3 Pharmacogenetics

A tendency towards higher C_max_/DW in *ABCB1* rs2032582 G/G + A/A+ G/A subjects compared to *ABCB1* T/T (*p univariate (p*_*uv*_ *)* = 0.056, *p multivariate* (*p*_*mv*_) = 0.049, β = 0.243, R^2^ = 0.067) (Table 4). Volunteers with the *ABCC2* rs2273697 A/A genotype showed a trend to higher C_max_/DW compared to the G/A genotype (*p*_*uv*_ = 0.087, *p*_*mv*_ = 0.048, β = 0.267, R^2^ = 0.067) (Table 4).

**TABLE 4:** Ezetimibe pharmacokinetic parameters based on genotypes or phenotypes.

	N	AUC_72h_/DW (h^a^ng^a^kg/mL^a^mg)	C_max_/DW (ng^a^kg/mL^a^mg)	t_max_ (h)
*ABCB1* (rs1128503)		*p =* 0.164	*p =* 0.245	*p =* 0.146
T/T	29	697.48 (281.49)	36.01 (16.98)	5.50 (0.75–6.00)
T/C	43	783.10 (291.38)	37.05 (19.68)	6.00 (1.75–6.50)
C/C	24	654.98 (261.32)	30.26 (15.14)	3.25 (1.50–6.38)
*ABCB1* (rs1045642)		*p =* 0.366	*p =* 0.555	*p =* 0.958
T/T	24	655.13 (232.91)	31.82 (11.61)	5.50 (0.75–6.38)
T/C	42	761.85 (284.24)	36.97 (18.73)	5.50 (1.25–6.00)
C/C	30	729.98 (316.49)	34.89 (20.74)	5.50 (1.50–6.50)
*ABCB1* (rs2032582)		*p =* 0.299	*p =* 0.056	*p =* 0.493
T/T	17	642.81 (280.73)	28.02 (13.69)	6.00 (1.75–6.50)
T/G + T/A	28	745.50 (268.91)	33.91 (16.28)	4.25 (1.50–6.00)
G/G + A/A+ G/A	50	746.37 (293.68)	38.25 (19.54)	5.50 (1.19–6.50)
*ABCC2* (rs2273697)		*p =* 0.137	*p =* 0.074	*p =* 0.995
G/G	68	714.18 (269.98)	34.93 (17.18)	5.50 (1.50–6.38)
G/A	25	715.89 (309.92)	32.73 (18.91)	5.50 (1.00–6.25)
A/A	3	1,052.81 (243.58)	56.59 (15.32)	6.00 (1.00–6.00)^a^
SLCO1B1		*p =* 0.221	*p =* 0.914	*p =* 0.373
NF	76	700.50 (271.62)	35.13 (18.90)	5.50 (1.25–6.00)
DF	19	830.65 (319.11)	34.54 (14.04)	6.00 (1.75–6.00)
PF	1	599.21	37.21	7.00
UGT1A1		*p =* 0.247	*p =* 0.407	*p =* 0.679
NM	44	685.06 (302.40)	34.25 (18.81)	5.75 (1.56–6.00)
IM	43	745.03 (258.16)	33.81 (13.20)	5.50 (1.00–6.00)
PM	8	820.20 (321.81)	44.26 (31.33)	5.75 (2.13–6.38)
*UGT1A3* (rs2008584)		*p =* 0.586	*p =* 0.163	*p =* 0.229
A/A	22	676.81 (264.31)	28.76 (11.78)	6.00 (1.69–6.50)
A/G	33	758.63 (310.61)	37.58 (18.99)	3.00 (1.00–6.00)
G/G	19	713.59 (308.52)	31.35 (11.25)	5.50 (2.00–6.50)
*UGT2B15* (rs1902023)		*p =* 0.400	*p =* 0.815	*p =* 0.742
A/A	16	782.84 (311.45)	33.18 (16.49)	6.00 (1.81–6.00)
A/C	47	681.01 (285.78)	31.77 (15.52)	5.50 (1.50–6.00)
C/C	17	737.18 (251.17)	33.22 (12.83)	6.00 (0.75–8.25)

Data are shown as mean (standard deviation) for normal distributions and median (Q25-Q75) for non-normal distributions. AUC: area under the curve. DW: dose/weight ratio. C_max_: maximum concentration. t_max_: time to C_max_. *p*-value of the group is shown.

^a^ It is not possible to generate quartiles, therefore, the range of the data is shown for this result.

There was a tendency towards higher AUC_72h_/DW in DF + PF subjects for SLCO1B1 compared to individuals who were SLCO1B1 NFs but it was not significant (*p*_*uv*_ = 0.107) (Table 4). A similar trend towards higher AUC_72h_/DW in subjects who were UGT1A1 IM + PM compared to NMs was observed but it did not reach statistical significance either (*p*_*uv*_ = 0.121) (Table 4). No differences or trends in pharmacokinetic parameters were observed considering the main candidate genes (*UGT1A3* and *UGT2B15*) (Table 4) and the remaining genes of metabolism and transport (Supplementary Table S1). There were no clinically relevant adverse events during the study.

## 4 Discussion

It is frequent for patients under treatment with statins not to reach the expected cholesterol levels. This is the reason for the emergence of combination therapies with different antihyperlipidemic drugs, such as ezetimibe, an inhibitor of intestinal uptake of cholesterol (Hirota et al., 2020; Gryn and Hegele, 2015). Pharmacogenetic information on ezetimibe is limited, and there are currently no clinical pharmacogenetic guidelines for this drug, although there are guidelines for other drugs such statins (Cooper-DeHoff et al., 2022). Therefore, the objective was to seek for pharmacogenetic biomarkers of pharmacokinetic variability in ezetimibe treatment.

Similar to previous works, men exhibited higher weight, height, and BMI than women (Zubiaur et al., 2021b; Soria-Chacartegui et al., 2023). The lack of AUC_72h_ and C_max_ differences between men and women after weight correction suggests that the lower weight of women was responsible for these differences. Results of t_max_ are consistent with the summary of product characteristics (Ministerio de Sanidad and Política Social e Igualdad, 2013). Due to the enterohepatic recirculation of this drug, it was not possible to obtain the half-life for most subjects using the sampling points collected during the clinical bioequivalence study. Therefore, this parameter was removed from the analysis as it was very difficult to interpret the data obtained. No differences between biogeographical origins in the pharmacokinetics of ezetimibe were found, which was in agreement with previous studies (Kosoglou et al., 2005). No differences were found between the administration of ezetimibe alone and the administration of ezetimibe plus simvastatin, as reported in the summary of product characteristics (Ministerio de Sanidad and Política Social e Igualdad, 2023).

Ezetimibe undergoes rapid and almost complete glucuronidation by UDP-glucuronosyltransferases (UGT) in enterocytes during its first passage, mainly by UGT1A1 enzyme, but also by UGT1A3 and UGT2B15 (Bae et al., 2011). In our research, a non-significant tendency towards higher AUC_72h_/DW in UGT1A1 IMs and PMs compared to NMs was observed. Bae *et al.* in 2011 found higher AUC_48h_ and C_max_ results in UGT1A1 PM healthy Korean male volunteers (Bae et al., 2011) compared to UGT1A1 NMs; however, Cai *et al.* in 2010 found no differences in mice (Cai et al., 2010). Thus, glucuronidation by UGT1A1 plays an important role in the overall metabolism of ezetimibe, but the impact of its genetic variation on ezetimibe pharmacokinetics seems not to be relevant. No differences were also observed for *UGT1A3* and *UGT2B15* genes. The characterization of the variants of these genes is not known in depth, so further studies, e.g., functional characterization and allele definition, would be needed to know the true involvement of these genes and other *UGTs*.

ABCC2 is an ATP-binding cassette transporter that is expressed in both the liver and intestine and is one of the transporters responsible for enterohepatic recirculation of ezetimibe (Oswald et al., 2011; de Waart et al., 2009). In addition, evidence from previous studies showed that ezetimibe is also a P-glycoprotein substrate, coded by *ABCB1* gene (Oswald et al., 2006). Despite these functions, information on the possible involvement of variants of these genes in the pharmacokinetics of ezetimibe is scarce and conflicting, with studies reporting no effect and others showing lower ezetimibe levels when ABCC2 and ABCB1 deficiency was present (Oswald et al., 2008; Oswald et al., 2010). In this study, a trend towards higher C_max_ in homozygous carriers of *ABCC2* (rs2273697) A allele and *ABCB1* (rs2032582) G/G + A/A+ G/A subjects was observed, that was confirmed in the multivariate analysis. No significant differences were observed in *ABCB1* rs1128503 and rs1045642 variants. Since *ABCC2* and *ABCB1* genes are functionally poorly characterized and widely distributed in the organism, the true implications of these results cannot be confirmed until functional studies of this gene and the involvement of its variants are performed. Regarding SLCO1B1 transporter, a non-significant trend of increased AUC_72h_ in volunteers with a DF phenotype compared to NF was observed. To date, only one study found changes in the pharmacokinetics of ezetimibe, observing higher exposure when the SLCO1B1 (also called OATP1B1) transporter had decreased function (Oswald et al., 2008).

### 4.1 Limitations

The small number of subjects included in this study was a limitation due to reduced statistical power. Since this was a study of healthy volunteers, the effect of variants on drug efficacy was not observed. The metabolite, ezetimibe glucuronide, was not measured in this study. Some of the genes that were analyzed, such as *ABCB1*, *ABCC2* and some *UGTs*, do not have well-characterized variant effects or defined alleles. In the case of the transporters, as they are expressed in different parts of the organism, it is more difficult to know whether a variant cause more or less function of the protein. Finally, because this is a candidate gene study with selected variants for each gene, we lose information about other variants that may also alter the kinetics of ezetimibe.

## 5 Conclusion

Pharmacogenetic information on ezetimibe is limited and there are currently no clinical pharmacogenetic guidelines for this drug. No association of metabolizing enzyme variants with ezetimibe pharmacokinetic parameters was found, but C_max_ was related with *ABCB1* rs2032582 and *ABCC2* rs2273697. Nevertheless, these results need to be validated in future studies.

## Future perspectives

In the future, new studies with a larger numbers of subjects should be carried out to confirm the results obtained in this article with regard to *ABCB1* and *ABCC2* and the rest of the genes for which no significant differences were obtained, and it would be an option to expand the variants studied for these genes. In addition, it would be interesting to analyze other genes that have been studied in relation to ezetimibe in previous articles, such as *NPC1L1*, other members of the *ABCG2* family such as *ABCG5* and *ABCG8*, or other *UGTs*.

## Data Availability

The original contributions presented in the study are publicly available. This data can be found here: repository of the Comunidad de Madrid, https://hdl.handle.net/20.500.12530/87955.

## References

[B1] Bae J. W. Choi C. I. Lee J. H. Jang C. G. Chung M. W. Lee S. Y. (2011). Effects of UDP-glucuronosyltransferase polymorphisms on the pharmacokinetics of ezetimibe in healthy subjects. Eur. J. Clin. Pharmacol. 67 (1), 39–45. 10.1007/s00228-010-0899-x 20865252

[B2] Birdwell K. A. Decker B. Barbarino J. M. Peterson J. F. Stein C. M. Sadee W. (2015). Clinical pharmacogenetics implementation Consortium (CPIC) guidelines for CYP3A5 genotype and tacrolimus dosing. Clin. Pharmacol. Ther. 98 (1), 19–24. 10.1002/cpt.113 25801146 PMC4481158

[B3] Bousman C. A. Stevenson J. M. Ramsey L. B. Sangkuhl K. Hicks J. K. Strawn J. R. (2023). Clinical pharmacogenetics implementation Consortium (CPIC) guideline for CYP2D6, CYP2C19, CYP2B6, SLC6A4, and HTR2A genotypes and serotonin reuptake inhibitor antidepressants. Clin. Pharmacol. Ther. 114 (1), 51–68. 10.1002/cpt.2903 37032427 PMC10564324

[B4] Boutari C. Karagiannis A. Athyros V. G. (2021). Rosuvastatin and ezetimibe for the treatment of dyslipidemia and hypercholesterolemia. Expert Rev. Cardiovasc Ther. 19 (7), 575–580. 10.1080/14779072.2021.1940959 34102931

[B5] Cai H. Nguyen N. Peterkin V. Yang Y. S. Hotz K. La Placa D. B. (2010). A humanized UGT1 mouse model expressing the UGT1A1*28 allele for assessing drug clearance by UGT1A1-dependent glucuronidation. Drug Metab. Dispos. Biol. Fate Chem. 38 (5), 879–886. 10.1124/dmd.109.030130 20124398 PMC2872941

[B6] Calderon-Ospina C. A. Hernández-Sómerson M. García A. M. Mejia A. Tamayo-Agudelo C. Laissue P. (2020). A pharmacogenomic dissection of a rosuvastatin-induced rhabdomyolysis case evokes the polygenic nature of adverse drug reactions. Pharmacogenomics Pers. Med. 13, 59–70. 10.2147/PGPM.S228709 PMC7060025 32184647

[B7] Campodónico D. M. Zubiaur P. Soria-Chacartegui P. Casajús A. Villapalos-García G. Navares-Gómez M. (2022). CYP2C8*3 and *4 define CYP2C8 phenotype: an approach with the substrate cinitapride. Clin. Transl. Sci. 15 (11), 2613–2624. 10.1111/cts.13386 36065758 PMC9652446

[B8] Committee for Medicinal Products for Human Use (CHMP) (2019). Ezetimibe tablet 10 mg product-specific bioequivalence guidance. Amsterdam, Netherlands: European Medicines Agency.

[B9] Cooper-DeHoff R. M. Niemi M. Ramsey L. B. Luzum J. A. Tarkiainen E. K. Straka R. J. (2022). The clinical pharmacogenetics implementation Consortium guideline for SLCO1B1, ABCG2, and CYP2C9 genotypes and statin-associated musculoskeletal symptoms. Clin. Pharmacol. Ther. 111 (5), 1007–1021. 10.1002/cpt.2557 35152405 PMC9035072

[B10] de Waart D. R. Vlaming M. L. H. Kunne C. Schinkel A. H. Oude Elferink R. P. J. (2009). Complex pharmacokinetic behavior of ezetimibe depends on abcc2, abcc3, and abcg2. Drug Metab. Dispos. Biol. Fate Chem. 37 (8), 1698–1702. 10.1124/dmd.108.026146 19443695

[B11] European Medicines Agency (2024). ICH E6 (R2) Good clinical practice - scientific guideline. Available at: https://www.ema.europa.eu/en/ich-e6-r2-good-clinical-practice-scientific-guideline.

[B12] Ferreira A. M. Marques da Silva P. (2017). Defining the place of ezetimibe/atorvastatin in the management of hyperlipidemia. Am. J. Cardiovasc Drugs Drugs Devices Interv. 17 (3), 169–181. 10.1007/s40256-016-0205-0 27943172

[B13] Gammal R. S. Court M. H. Haidar C. E. Iwuchukwu O. F. Gaur A. H. Alvarellos M. (2016). Clinical pharmacogenetics implementation Consortium (CPIC) guideline for UGT1A1 and atazanavir prescribing. Clin. Pharmacol. Ther. 99 (4), 363–369. 10.1002/cpt.269 26417955 PMC4785051

[B14] Gryn S. E. Hegele R. A. (2015). Ezetimibe plus simvastatin for the treatment of hypercholesterolemia. Expert Opin. Pharmacother. 16 (8), 1255–1262. 10.1517/14656566.2015.1041504 25920750

[B15] Hegele R. A. Guy J. Ban M. R. Wang J. (2005). NPC1L1 haplotype is associated with inter-individual variation in plasma low-density lipoprotein response to ezetimibe. Lipids Health Dis. 4, 16. 10.1186/1476-511X-4-16 16098225 PMC1198250

[B16] Hirota T. Fujita Y. Ieiri I. (2020). An updated review of pharmacokinetic drug interactions and pharmacogenetics of statins. Expert Opin. Drug Metab. Toxicol. 16 (9), 809–822. 10.1080/17425255.2020.1801634 32729746

[B17] Jeu L. Cheng J. W. M. (2003). Pharmacology and therapeutics of ezetimibe (SCH 58235), a cholesterol-absorption inhibitor. Clin. Ther. 25 (9), 2352–2387. 10.1016/s0149-2918(03)80281-3 14604738

[B18] Kei A. A. Filippatos T. D. Elisaf M. S. (2016). The safety of ezetimibe and simvastatin combination for the treatment of hypercholesterolemia. Expert Opin. Drug Saf. 15 (4), 559–569. 10.1517/14740338.2016.1157164 26898906

[B19] Kosoglou T. Statkevich P. Johnson-Levonas A. O. Paolini J. F. Bergman A. J. Alton K. B. (2005). Ezetimibe: a review of its metabolism, pharmacokinetics and drug interactions. Clin. Pharmacokinet. 44 (5), 467–494. 10.2165/00003088-200544050-00002 15871634

[B20] Ministerio de Sanidad, Política Social e Igualdad (2013). “Agencia Española de Medicamentos y Productos Sanitarios,” in Ficha técnica ezetimiba sandoz 10 mg comprimidos EFG.

[B21] Ministerio de Sanidad, Política Social e Igualdad (2023). “Agencia Española de Medicamentos y Productos Sanitarios,” in Ficha técnica ezetimiba/simvastatina stada 10 mg/20 mg comprimidos EFG.

[B22] Oswald S. Haenisch S. Fricke C. Sudhop T. Remmler C. Giessmann T. (2006). Intestinal expression of P-glycoprotein (ABCB1), multidrug resistance associated protein 2 (ABCC2), and uridine diphosphate–glucuronosyltransferase 1A1 predicts the disposition and modulates the effects of the cholesterol absorption inhibitor ezetimibe in humans. Clin. Pharmacol. Ther. 79 (3), 206–217. 10.1016/j.clpt.2005.11.004 16513445

[B23] Oswald S. König J. Lütjohann D. Giessmann T. Kroemer H. K. Rimmbach C. (2008). Disposition of ezetimibe is influenced by polymorphisms of the hepatic uptake carrier OATP1B1. Pharmacogenet Genomics. 18 (7), 559–568. 10.1097/FPC.0b013e3282fe9a2c 18551036

[B24] Oswald S. May K. Rosin J. Lütjohann D. Siegmund W. (2010). Synergistic influence of Abcb1 and Abcc2 on disposition and sterol lowering effects of ezetimibe in rats. J. Pharm. Sci. 99 (1), 422–429. 10.1002/jps.21821 19504475

[B25] Oswald S. Nassif A. Modess C. Keiser M. Ulrich A. Runge D. (2011). Drug interactions between the immunosuppressant tacrolimus and the cholesterol absorption inhibitor ezetimibe in healthy volunteers. Clin. Pharmacol. Ther. 89 (4), 524–528. 10.1038/clpt.2011.4 21368751

[B26] Schmitz G. Schmitz-Mądry A. Ugocsai P. (2007). Pharmacogenetics and pharmacogenomics of cholesterol-lowering therapy. Curr. Opin. Lipidol. 18 (2), 164–173. 10.1097/MOL.0b013e3280555083 17353665

[B27] Soria-Chacartegui P. Zubiaur P. Ochoa D. Villapalos-García G. Román M. Matas M. (2023). Genetic variation in CYP2D6 and SLC22A1 affects amlodipine pharmacokinetics and safety. Pharmaceutics 15 (2), 404. 10.3390/pharmaceutics15020404 36839726 PMC9959242

[B28] Soulele K. Karalis V. (2019). On the population pharmacokinetics and the enterohepatic recirculation of total ezetimibe. Xenobiotica 49 (4), 446–456. 10.1080/00498254.2018.1463117 29629619

[B29] Tada H. Okada H. Nomura A. Takamura M. Kawashiri M. A. (2020). Beneficial effect of ezetimibe-atorvastatin combination therapy in patients with a mutation in ABCG5 or ABCG8 gene. Lipids Health Dis. 19 (1), 3. 10.1186/s12944-019-1183-4 31901240 PMC6942309

[B30] World Medical Association Declaration of Helsinki (2013). Ethical principles for medical research involving human subjects. JAMA 310 (20), 2191. 10.1001/jama.2013.281053 24141714

[B31] Zambrano T. Saavedra N. Lanas F. Caamaño J. Salazar L. A. (2015). Efficacy of ezetimibe is not related to NPC1L1 gene polymorphisms in a pilot study of Chilean hypercholesterolemic subjects. Mol. Diagn Ther. 19 (1), 45–52. 10.1007/s40291-014-0128-x 25589339

[B32] Zhan S. Tang M. Liu F. Xia P. Shu M. Wu X. (2018). “Ezetimibe for the prevention of cardiovascular disease and all-cause mortality events,” in Cochrane database syst rev. Editor Heart Group C. Available at: https://doi.wiley.com/10.1002/14651858.CD012502.pub2 (Accessed December 22, 2022). 10.1002/14651858.CD012502.pub2 PMC6516816 30480766

[B33] Zubiaur P. Benedicto M. D. Villapalos-García G. Navares-Gómez M. Mejía-Abril G. Román M. (2021b). SLCO1B1 phenotype and CYP3A5 polymorphism significantly affect atorvastatin bioavailability. J. Pers. Med. 11 (3), 204. 10.3390/jpm11030204 33805706 PMC7999651

[B34] Zubiaur P. Mejía-Abril G. Navares-Gómez M. Villapalos-García G. Soria-Chacartegui P. Saiz-Rodríguez M. (2021a). PriME-PGx: La Princesa university hospital multidisciplinary initiative for the implementation of pharmacogenetics. J. Clin. Med. 10 (17), 3772. 10.3390/jcm10173772 34501219 PMC8432257

